# Serum 3-Nitrotyrosine in the Cardiovascular Disease of Patients with Systemic Lupus Erythematosus

**DOI:** 10.3390/antiox14060739

**Published:** 2025-06-16

**Authors:** Juan C. Quevedo-Abeledo, Marta Hernández-Díaz, María García-González, Fuensanta Gómez-Bernal, Cristina Almeida-Santiago, Elena Heras-Recuero, Antonia de Vera-González, Alejandra González-Delgado, Pedro Abreu-González, Beatriz Tejera-Segura, Candelaria Martín-González, Miguel Á. González-Gay, Iván Ferraz-Amaro

**Affiliations:** 1 Division of Rheumatology, Hospital Doctor Negrín, 35010 Las Palmas de Gran Canaria, Spain; quevedojcarlos@yahoo.es (J.C.Q.-A.); almeidasantiago.cristina@gmail.com (C.A.-S.); 2 Division of Rheumatology, Hospital Universitario de Canarias, 38320 San Cristóbal de La Laguna, Spain; martahediaz@gmail.com (M.H.-D.); margagon23@hotmail.com (M.G.-G.); 3 Division of Central Laboratory, Hospital Universitario de Canarias, 38320 San Cristóbal de La Laguna, Spain; fuensanta95@gmail.com (F.G.-B.); adeverag@gmail.com (A.d.V.-G.); alejandra.gd88@gmail.com (A.G.-D.); 4 Division of Rheumatology, IIS-Fundación Jiménez Díaz, 28040 Madrid, Spain; 3bheraselena@gmail.com; 5 Unit of Physiology, Department of Basic Medical Sciences, University of La Laguna (ULL), 38200 San Cristóbal de La Laguna, Spain; pabreu@ull.edu.es; 6 Division of Rheumatology, Hospital Insular, 35016 Las Palmas de Gran Canaria, Spain; btejerasegura@gmail.com; 7 Department of Internal Medicine, University of La Laguna (ULL), 38200 San Cristóbal de La Laguna, Spain; mmartgon@ull.edu.es; 8 Department of Medicine and Psychiatry, University of Cantabria, 39005 Santander, Spain

**Keywords:** 3-nitrotyrosine, systemic lupus erythematosus, disease damage, cardiovascular disease

## Abstract

3-Nitrotyrosine (3-NT) is a product of tyrosine nitration mediated by reactive nitrogen species such as peroxynitrite anion and nitrogen dioxide. It serves as an indicator of inflammation, cell damage, and nitric oxide production. Systemic lupus erythematosus (SLE) is an autoimmune disorder characterized by multisystem involvement and increased oxidative stress. Notably, cardiovascular (CV) disease has emerged as the leading cause of mortality among SLE patients. Our objective was to investigate the association between serum 3-NT levels and a wide range of disease characteristics in patients with SLE, with a particular emphasis on CV comorbidity. A total of 214 patients with SLE were enrolled. The serum levels of 3-NT as well as the activity (SLEDAI) and damage index (SLICC-SDI) scores, full lipid profile, insulin resistance indices, and carotid subclinical atherosclerosis were assessed. Multivariable linear regression analysis was carried out to study the relationship between 3-NT and clinical and laboratory disease characteristics, especially focusing on CV comorbidities. Except for body mass index, which showed a significant positive correlation, the demographic data and traditional CV risk factors did not correlate with 3-NT. After multivariable adjustments, several disease characteristics, including the disease duration, activity and damage indices, and autoantibody profile, showed significant positive associations with 3-NT. Regarding CV characteristics, several lipid profile molecules showed significant relationships with 3-NT. This was not the case for insulin resistance and subclinical atherosclerosis. Remarkably, patients with a high CV risk by SCORE2 showed higher 3-NT values compared to those with a low risk, although after the multivariable adjustment, this relationship was attenuated (but still showed a trend). In conclusion, serum 3-NT levels demonstrated significant positive correlations with multiple disease characteristics, including the disease activity and damage and the autoantibody profile. The lipid pattern in the SLE subjects also significantly and independently correlated with the 3-NT values. Our findings highlight the pathophysiological role of 3-NT specifically, and peroxidation in general, in patients with SLE.

## 1. Introduction

3-Nitrotyrosine (3-NT) is a post-translational protein modification resulting from the action of nitrating agents, which leads to the addition of a nitro (−NO2) group to tyrosine residues, a process known as protein tyrosine nitration [1]. This modification can potentially alter the protein structure and function, contributing to cellular dysfunction in various disease states, and it is considered a specific marker of oxidative damage mediated by peroxynitrite. Numerous reports have shown the presence of 3-NT in various pathologic conditions [2]. For example, elevated levels of 3-NT have been reported in various human pathologies, such as atherosclerosis and cardiovascular disease (CVD) [3], neurodegenerative diseases like multiple sclerosis, Alzheimer’s disease or Parkinson’s disease [4], and lung diseases [5]. The peroxynitrite-mediated oxidation and nitration of biomolecules are hypothesized to contribute to the development of autoimmune diseases. The release of these modified proteins may trigger an immune response, leading to the production of antibodies against self-proteins. Specifically, tyrosine-nitrated proteins can function as neoantigens, potentially inducing the generation of autoantibodies against native proteins in various autoimmune disorders [1]. This mechanism suggests a link between oxidative stress, protein modification, and the breakdown of immune tolerance in autoimmune pathogenesis.

Systemic lupus erythematosus (SLE) is a chronic autoimmune disorder with a heterogeneous clinical presentation and the potential to involve multiple organ systems. The clinical manifestations range from mild cutaneous and articular symptoms to severe, life-threatening complications affecting the renal, hematological, or central nervous systems. Patients with SLE have a markedly increased risk of premature atherosclerosis, and cardiovascular disease (CVD) remains the leading cause of mortality in this population [6].

Oxidative stress has been observed to be increased in SLE [7,8]. This augmented oxidative stress is believed to be involved in causing inflammatory and cellular defects in the pathogenesis of SLE, leading to immune system dysregulation and various disease manifestations [9]. In this regard, a range of inflammatory and cellular markers, notably oxidative modifications to proteins, lipids, and DNA, play a crucial role in disrupting immune system homeostasis. This disruption can precipitate a vigorous autoimmune response, mediated through molecular pathways such as increased NETosis, mTOR pathway activation, and skewed T-cell differentiation [8,10,11].

In this study, we measured 3-NT levels in a well-characterized cohort of patients with SLE. These patients were characterized not only in terms of their disease features but also in aspects related to CVD, including a comprehensive lipid profile, insulin resistance indices, and the presence of subclinical atherosclerosis. Consequently, our aim was to investigate how disease characteristics, with a particular focus on those related to CVD, are associated with 3-NT levels. By examining these associations, we aim to better understand the potential role of oxidative stress in the CV complications often observed in SLE.

## 2. Materials and Methods

### 2.1. Study Participants

This cross-sectional study included 214 patients diagnosed with SLE. All participants were aged 18 years or older, had a clinical diagnosis of SLE, and fulfilled at least four of the American College of Rheumatology (ACR) classification criteria for the disease [12]. They had been diagnosed by rheumatologists, were regularly followed up with in rheumatology outpatient clinics, and had a disease duration exceeding 1 year. Historical data were available in their medical records, and they were willing to undergo the specific study procedures. Participation was allowed for patients taking prednisone, at an equivalent dose of ≤10 mg/day, as glucocorticoids are often used in the treatment of SLE. The exclusion criteria included the presence of other autoimmune diseases, severe renal impairment (estimated glomerular filtration rate < 30 mL/min/1.73 m^2^), an active infection requiring systemic antibiotic or antiviral treatment, a known history of malignancy within the past 5 years, pregnancy or breastfeeding, and an inability to understand or comply with the study procedures. The research was carried out in accordance with the Declaration of Helsinki. The study protocol was approved by the Institutional Review Committee at Hospital Universitario de Canarias and at Hospital Universitario Doctor Negrín (both in Spain), and all subjects provided informed written consent (Approval Number CHUC−2023−48).

### 2.2. Data Collection

The participants underwent a comprehensive CV risk evaluation through standardized protocols. The baseline assessments included the completion of validated questionnaires detailing the medical history, medication regimens, and lifestyle factors. The clinicians performed physical examinations with a standardized measurement of the anthropometric parameters (weight, height, body mass index) and abdominal circumference. Blood pressure measurements were obtained in the supine position using calibrated equipment and following established protocols. The participants’ self-reported smoking status and antihypertensive medication use were recorded through structured interviews. The researchers conducted systematic reviews of electronic health records to confirm the clinical diagnoses and validate the prescribed pharmaceutical treatments. All of the data collection procedures followed quality-controlled protocols to ensure consistency across the measurements.

The SLE disease activity and damage were evaluated using the Systemic Lupus Erythematosus Disease Activity Index −2000 (SLEDAI−2K) [13] and the Systemic Lupus International Collaborating Clinics/American College of Rheumatology (SLICC/ACR) Damage Index -SDI [14], respectively. For the study proposal, the SLEDAI−2k index was divided into none (0 points), mild (1–5 points), moderate (6–10 points), high (11–19), and very high activity (>20), as previously described [15]. Definitions of Remission in SLE (DORIS) was based on the absence of clinical disease activity, as measured by the clinical SLEDAI−2K = 0 and physician global assessment (PGA) <0.5. The patient may be on antimalarials, low-dose glucocorticoids (e.g., prednisone ≤5 mg/day), and/or maintenance doses of immunosuppressive therapies [16]. Similarly, the Lupus Low Disease Activity State (LLDAS) accepts a SLEDAI−2K ≤4 with no activity from the major organ systems, no new clinical activity compared with the previous assessment, a PGA of ≤1, a prednisone dose of ≤7.5 mg/day, and maintenance doses of antimalarials and immunosuppressive therapies [17]. The Systematic Coronary Risk Evaluation−2 (SCORE2) CV risk tool was assessed, as previously described, using age, gender, smoking status, systolic blood pressure, and non-HDL-cholesterol [18]. The SCORE2 estimates an individual’s 10-year risk of fatal and non-fatal CV disease events in individuals aged 40 to 69 years. For healthy people aged ≥70 years, the SCORE2-OP (older persons) algorithm estimates the 5-year and 10-year fatal and nonfatal CV disease events.

### 2.3. Laboratory Assessments

The serum cholesterol, triglycerides, and HDL-cholesterol were determined using enzymatic colorimetric assays (Roche Farma, Madrid, Spain). The serum lipoproteins were measured by a quantitative immunoturbidimetric method (Roche Farma, Madrid, Spain). The range for cholesterol was 0.08 to 20.7 mmol/L (intra-assay coefficient of variation: 0.3%), for triglycerides 4 to 1000 mg/dl (intra-assay coefficient of variation: 1.8%), and for HDL-cholesterol 3 to 120 mg/dl (intra-assay coefficient of variation: 0.9%). The atherogenic index was calculated as the total cholesterol to HDL-cholesterol ratio according to the Castelli formula, and the LDL-cholesterol was estimated using the Friedewald equation. The high-sensitivity C-reactive protein (hs-CRP) levels were measured using a high-sensitivity immunoassay. 3-NT was measured using an enzyme-linked immunosorbent assay (ELISA) kit from Invitrogen (Catalogue number ELL008, Thermo Fisher Scientific Inc., Waltham, MA, USA). Both the intra- and inter-coefficients of variability for this assay were maintained below 10%.

The Homeostatic Model Assessment (HOMA) method was utilized to evaluate the insulin resistance and related metabolic parameters. Specifically, the updated HOMA2 model was employed, which allows for the estimation of insulin sensitivity (%S) and β-cell function (%B) using fasting plasma glucose, insulin, or C-peptide concentrations. This model accommodates a broader range of values, including insulin levels between 1 and 2200 pmol/L and glucose concentrations from 1 to 25 mmol/L. In this study, insulin resistance (IR) and %S were calculated based on fasting serum insulin levels, while %B was derived using serum C-peptide concentrations due to its reliability as a marker of β-cell secretion. The HOMA2 computer model provided results for insulin sensitivity expressed as HOMA2-%S, with 100% representing normal sensitivity. The insulin resistance was quantified using HOMA2-IR, which is calculated as the reciprocal of %S. Insulin (Architect Abbott, 2000I) and C peptide (Immulite 2000, Siemens) were determined by chemiluminescent immunometric assays.

### 2.4. Carotid Ultrasound Assessment

Carotid ultrasonography was conducted to assess the intima–media thickness (cIMT) of the common carotid artery. The evaluation was performed using an EsaoteMylab 70 ultrasound device (Genova, Italy) fitted with a linear probe operating at a 7–12 MHz frequency. The measurements were obtained through the automated Quality Intima Media Thickness in real-time (QIMT) software system utilizing radiofrequency-based technology developed by Esaote (Maastricht, Holland). The evaluation followed the Mannheim consensus recommendations, which outline how to identify plaques in the extracranial carotid arteries that are accessible for assessment [19]. A plaque was defined as a distinct protrusion into the arterial lumen where the carotid intima–media thickness (cIMT) measured more than 1.5 mm. Furthermore, this protrusion had to be at least 50% greater than the surrounding cIMT or cause a narrowing of the arterial lumen by more than 0.5 mm [19].

### 2.5. Statistical Analysis

The demographic and clinical characteristics in the patients with SLE and the controls were described as the mean ± standard deviation (SD) or percentages for categorical variables. For the non-normally distributed continuous variables, the data were expressed as the median and interquartile range (IQR). Non-normal distribution was assessed using the Shapiro–Wilk test. The relationship of disease characteristics with circulating 3-NT was evaluated by multivariable linear regression analysis. Confounders were selected from the demographics and disease-related data if they had a relationship with 3-NT with a *p* value less than 0.20 [20]. Given the non-normal distribution of the 3-NT variable in the regression analyses, its inverse multiplied by 1000 was used. The results, expressed as beta coefficients, should be interpreted according to this transformation (1/(ng/mL) × 1000. All of the analyses used a 5% two-sided significance level and were performed using Stata software, version 17/SE (StataCorp, College Station, TX, USA). The *p* values <0.05 were considered statistically significant.

## 3. Results

### 3.1. Demographics and Disease-Related Data of Systemic Lupus Erythematosus Patients

The median serum concentration of 3-nitrotyrosine (3-NT) among individuals with SLE was 5.9 ng/mL, with an interquartile range of 4.7 to 8.0 ng/mL. Table 1 provides an overview of the main features of the 214 participants enrolled in the study. Most of the participants were female (91%), with an average age of 51 ± 12 years. The mean BMI was 28 kg/m^2^ (SD ± 6), and the mean waist circumference was 91 ± 15 cm. Traditional CV risk factors were present, including current smoking in 19% of the subjects, hypertension in 38%, and obesity in 27%. Statin use was reported in 29% of the cohort, while 28% were on aspirin therapy. Carotid ultrasounds detected plaques in 44% of the patients, and the mean cIMT was 657 ± 144 microns. Further details on the lipid parameters and insulin resistance measures are available in Table 1.

The mean duration of SLE among the participants was 18 years, with a standard deviation of 11 years. According to the SLEDAI score, 37% of the patients exhibited no disease activity, while 52% had mild activity and 11% showed moderate to high activity. The median SDI was 1 (IQR 0–1), and over half of the patients (53%) had an SDI score of at least 1. Prednisone was being used by 35% of the cohort, with a median daily dose of 5 mg (IQR 2.5–5 mg). At the time of enrollment, 75% of the patients were positive for anti-DNA antibodies and 72% for ENA antibodies, with anti-SSA being the most common (detected in 35%). Hydroxychloroquine had been prescribed to 72% of the patients. Other disease-modifying antirheumatic drugs, such as methotrexate (13%) and mycophenolate mofetil (12%), were used less frequently. Further details regarding the SLE-specific characteristics are presented in Table 1.

### 3.2. Relationship Demographics and Disease-Related Data with 3-Nitrotyrosine

Regarding the demographic characteristics, only BMI—whether analyzed as a continuous variable or categorized as ≥30 kg/m^2^—showed a significant association with 3-NT levels. Notably, classic CV risk factors such as smoking, diabetes, and hypertension, as well as the use of statins or aspirin, were not associated with 3-NT values (Table 2).

**Figure 1:**
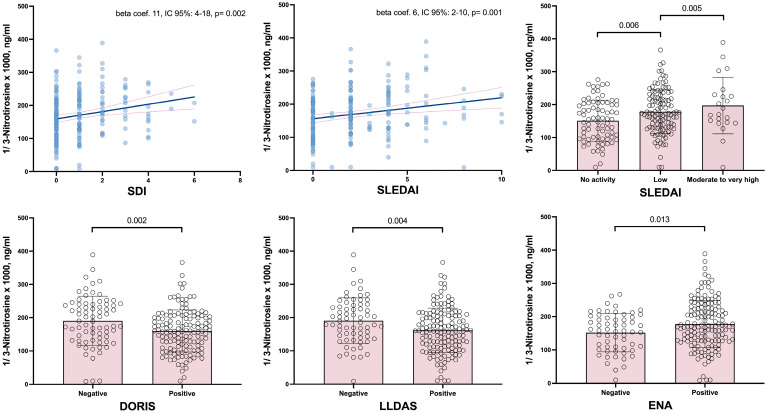
Relation of SDI and SLEDAI scores and DORIS, LLDAS, and ENA to 3-NT serum levels. SDI: damage index; SLEDAI: Systemic Lupus Erythematosus Disease Activity Index; LLDAS: Low Lupus Disease Activity State; DORIS: Definitions of Remission In SLE; ENA: extractable autoantibodies.

On the other hand, numerous associations were observed between the disease characteristics and the serum levels of 3-NT. In this regard, the disease duration, disease-related damage, and disease activity were significantly associated with the serum levels of 3-NT. Specifically, the SDI, both as a continuous variable and when categorized as 0 or ≥1, was associated with higher 3-NT levels. Similarly, the SLEDAI showed that patients with mild or moderate and high disease activity had higher 3-NT values compared to those in remission. Both the SLEDAI−2K and SDI relationships remained significant after multivariate adjustment (Table 2 and Figure 1). Since the SLEDAI−2K and SDI scores are constructed by summing different items corresponding to various domains or organ manifestations, we have broken down, for both calculators, the relationship of their respective items with 3-NT. In the case of SDI, the domains that showed statistical significance with 3-NT values were the CV, articular, and skin domains (Supplementary Table S1). For the SLEDAI−2K, the significant items related to 3-NT were a rash and an elevated anti-DNA (Supplementary Table S2). Furthermore, patients who met the criteria for remission or low disease activity, as defined by the DORIS or LLDAS, exhibited significantly inferior 3-NT levels compared to those who did not achieve these disease states (Table 2 and Figure 1).

Regarding the autoantibody profiles, positive associations with 3-NT were also observed. After adjusting for covariates, the patients who were positive for extractable nuclear antibodies (ENAs), anti-RNP, anti-ribosome, and anti-nucleosome antibodies exhibited significantly higher circulating 3-NT levels. For anti-dsDNA and anti-SSB, although statistical significance was not reached, a trend was noted. Remarkably, positivity for lupus anticoagulant, but not anti-cardiolipin IgG/IgM and anti-β2-glycoprotein I IgG/IgM, showed a significant relationship with 3-NT, although in this case the association was negative. Finally, none of the therapies used by the patients demonstrated a significant association with 3-NT levels (Table 2 and Figure 1).

### 3.3. Multivariable Analysis of Cardiovascular Disease-Related Factors’ Association with 3-Nitrotyrosine

The lipid profile was prominently associated with 3-NT values (Table 3). In this regard, after the multivariable adjustment, the triglycerides, non-HDL cholesterol, apolipoprotein B, LDL:HDL and ApoB:ApoA1 ratios, and the atherogenic index showed significant positive associations with 3-NT levels. In contrast, the insulin resistance indices did not demonstrate significant relations to 3-NT values. Similarly, the presence of carotid plaque and cIMT was not significantly associated with 3-NT levels. Notably, in the univariable analysis, patients with a high-risk SCORE2 exhibited significantly higher values compared to those at low risk. Although this significance was lost after the multivariable adjustment, a clear trend in this direction was observed (Table 3).

## 4. Discussion

Our study is the first to evaluate 3-NT in patients with SLE, specifically investigating its relationship with CV disease-related characteristics, including a comprehensive lipid profile, insulin resistance indices, and subclinical atherosclerosis. According to our results, 3-NT, a marker of protein oxidative stress, is associated not only with various disease characteristics but also with the abnormal lipid profile typically observed in these patients. This novel approach offers insights into the complex interplay between oxidative stress, lipid metabolism, and CV risk in SLE patients.

A substantial body of evidence, derived from numerous studies, indicates that patients with SLE exhibit elevated 3-NT levels compared to controls [21,22,23,24,25,26]. Additionally, previous research has explored the role of 3-NT in SLE. For instance, patients with active lupus nephritis have been found to have higher serum 3-NT levels than those without renal disease [21]. Similarly, in a study of 25 patients with SLE, those with high SLEDAI scores or elevated anti-dsDNA antibody levels exhibited increased oxidation compared with patients with low SLEDAI scores or low antibody levels [24]. Another study of 26 SLE patients also found a significant correlation between 3-NT and the SLEDAI, with renal variables within the SLEDAI showing the strongest correlation with serum 3-NT levels [22]. Our study includes the largest and most comprehensive cohort of SLE patients to date in which 3-NT levels have been analyzed. Our findings corroborate the previously mentioned reports by demonstrating a clear association between 3-NT levels and disease activity. This association was further confirmed in the multivariable analysis. Moreover, we describe a positive relationship between 3-NT and disease-related damage, as well as an inverse relationship with states of remission and low disease activity. These findings robustly confirm that 3-NT is positively associated with states of increased disease activity and damage.

We also found a positive association between 3-NT and the disease’s autoantibody profile. Prior descriptions have detailed how the presence of a nitro group on a tyrosine residue can elicit an immunological response [1]. These data indicate a potential role for nitrated epitopes of autologous proteins in the development of autoantibodies in SLE, potentially influencing the disease’s etiopathogenesis. Additionally, and surprisingly, we found a relationship between 3-NT and lupus anticoagulant, but in this case, the relationship was negative. We do not have a precise explanation for this. It is possible that patients with lupus anticoagulant more frequently take aspirin, which could have a negative effect on 3-nitrotyrosine. In this regard, aspirin has antioxidant properties and can reduce oxidative stress, which could potentially lower the levels of 3-NT. Since 3-NT is a marker of oxidative stress and nitrative damage, the aspirin use could dampen the oxidative environment in these patients, resulting in lower levels of 3-NT. In some patients with lupus anticoagulant, thrombotic processes may predominate over inflammatory ones. This could lead to a reduction in systemic inflammation or oxidative stress, which might otherwise decrease 3-NT levels.

A correlation between 3-NT and elevated levels of several lipid profile components was observed. Therefore, 3-NT appeared to be associated with a deleterious lipid profile in terms of the CV risk in patients with SLE. It is known that dyslipidemia can contribute to increased oxidative stress [27]. In this sense, oxidized LDL cholesterol is particularly damaging to blood vessel walls and has been strongly associated with angiographically documented coronary artery disease [28]. In addition, lipoproteins from SLE patients are more oxidized and have greater oxidative potential, which may contribute to accelerated atherosclerosis [29]. Based on our findings, we suggest that 3-NT may contribute to dyslipidemia in SLE and potentially increase the CV risk in these patients.

These findings regarding the association between 3-NT and both disease activity and damage, as well as its relationship with the autoantibody profile, suggest a clear link between the oxidative stress and inflammation marker 3-NT and major clinical outcomes in patients with SLE. We hypothesize that the overproduction of reactive nitrogen species in the pathogenesis of human SLE leads to increased nitration of tyrosine residues on serum proteins and the formation of 3-NT. Importantly, 3-NT has been proven to bind more strongly to SLE immunoglobulins than to other antigens such as dsDNA or native poly L-tyrosine, suggesting the formation of immunogenic nitrotyrosine-bearing epitopes [26]. These modified self-proteins may activate autoreactive T cells and contribute to the breakdown of immunological tolerance, a hallmark of SLE. Furthermore, we believe that the persistent elevation of 3-NT may indicate either ongoing or recurrent bursts of protein nitration, with inefficient repair mechanisms in SLE patients. Our finding of 3-NT serum levels’ association with increased disease activity, nephritis, and CV disease in SLE supports its role as a biomarker linking oxidative stress and inflammation to major clinical outcomes. The formation of immune complexes between SLE IgG and nitrated proteins further implicates 3-NT in the amplification of autoimmune responses and organ damage. Thus, 3-NT not only reflects the burden of oxidative stress and inflammation but may also actively participate in the pathogenesis and progression of critical clinical manifestations in SLE.

In our study, patients classified as high-risk according to the SCORE2 algorithm exhibited significantly elevated 3-NT levels compared to those in the low-risk category. Although this association was attenuated following multivariable adjustment, a notable trend persisted. The observed relationship between 3-NT levels and the SCORE2, a composite measure integrating multiple CV risk factors predictive of CV events, underscores the potential significance of 3-NT as a marker of CV risk in SLE patients. This finding suggests that 3-NT may reflect the cumulative impact of various CV risk factors in SLE, potentially serving as an integrative biomarker of oxidative stress and CV risk. These findings may align with the concept that protein nitration within the arterial wall has been described as reducing endothelial cell adhesion, leading to endothelial denudation, thrombus formation, and phenotypic changes in smooth muscle cells [30]. These processes may collectively contribute to the rupture of atherosclerotic plaques, subsequent thrombus formation, and the occurrence of myocardial infarctions and strokes. Nevertheless, further investigation into the mechanistic links between oxidative stress, as measured by 3-NT, and CV risk in SLE is warranted.

Despite this, we did not observe an association between 3-NT levels and the presence of carotid plaque or cIMT measurements. Atherosclerosis is a chronic process in which CV risk factors play a central role. We believe that the cross-sectional nature of our study, along with the potential confounding effect of traditional CV risk factors, may have limited our ability to detect a relationship between 3-NT and markers of subclinical carotid atherosclerosis.

We acknowledge that we did not include a control group. However, the purpose of our study was not to compare 3-NT levels between controls and SLE patients, but rather to explore the relationship between this molecule and disease characteristics. Additionally, as previously mentioned, the differences in 3-NT serum levels between SLE patients and controls have been thoroughly explored in previous studies. The cross-sectional nature of our work prevents inferring causality, for which prospective studies would be required. In this regard, this cross-sectional design prevents us from drawing conclusions regarding the effect of disease therapies on 3-NT.

## 5. Conclusions

In conclusion, by analyzing 3-NT levels in relation to CV parameters, our study enhances the understanding of the mechanisms behind the elevated CV risk observed in SLE. Additionally, the relationship between 3-NT and lipid profile abnormalities points to a potential connection between oxidative stress and dyslipidemia in SLE, which may play a role in the accelerated atherosclerosis seen in these patients.

## Figures and Tables

**Table 1:** Characteristics of systemic lupus erythematosus patients.

	SLE patients
	(*n* = 214)
3-Nitrotyrosine, ng/mL	5.9 (4.7–8.0)
Age, years	51 ± 12
Female, *n* (%)	194 (91)
Body mass index, kg/m^2^	28 ± 6
Abdominal circumference, cm	91 ± 15
Waist circumference, cm	100 ± 15
Waist to hip ratio	0.92 ± 0.33
Cardiovascular co-morbidity	
Smoking, *n* (%)	40 (19)
Diabetes, *n* (%)	11 (5)
Hypertension, *n* (%)	82 (38)
Obesity, *n* (%)	58 (27)
Metabolic syndrome, *n* (%)	92 (44)
Statins, *n* (%)	61 (29)
Aspirin, *n* (%)	59 (28)
SCORE2, %	2.1 (1.1–3.8)
SCORE2 categories	
Low risk	171 (80)
Moderate risk	28 (13)
High risk	15 (7)
Carotid ultrasound	
Carotid intima–media thickness, microns	657 ± 144
Carotid plaque, *n* (%)	69 (44)
Laboratory data	
CRP, mg/dL	1.86 (0.8–4.14)
Cholesterol, mg/dL	183 ± 40
Triglycerides, mg/dL	112 ± 64
HDL-cholesterol, mg/dL	55 ± 15
LDL-cholesterol, mg/dL	106 ± 33
LDL:HDL-cholesterol ratio	2.06 ± 0.82
Non-HDL-cholesterol, mg/dL	128 ± 38
Lipoprotein A, mg/dL	32 (11–85)
Apolipoprotein A1, mg/dL	157 ± 28
Apolipoprotein B, mg/dL	87 ± 23
Apo B:Apo A1 ratio	0.57 ± 0.17
Atherogenic index	3.52 ± 1.09
Insulin resistance indices	
Glucose, mg/dL	92 ± 17
Insulin, µU/mL	14 ± 17
C-peptide, ng/mL	2.4 ± 1.69
HOMA2-IR	1.10 (0.70–1.80)
HOMA2-S%	93 (5–150)
HOMA2-B%-C-peptide	122 (95–153)
SLE-related data	
Disease duration, years	18 ± 11
CRP, mg/dL	1.9 (0.8–4.1)
SLICC-DI	1 (0–1)
SLICC-DI >=1, *n* (%)	113 (53)
SLEDAI−2K	2 (0–4)
SLEDAI categories, *n* (%)	
No activity, *n* (%)	80 (37)
Mild, *n* (%)	111 (52)
Moderate to high, *n* (%)	23 (11)
DORIS, *n* (%)	139 (65)
LLDAS, *n* (%)	146 (68)
Auto-antibody profile	
Anti-DNA positive, *n* (%)	160 (75)
Anti-ENA positive, *n* (%)	151 (72)
Anti-SSA, *n* (%)	75 (35)
Anti-SSB, *n* (%)	33 (15)
Anti-Sm, *n* (%)	39 (18)
Anti-RNP, *n* (%)	63 (30)
Anti-ribosome	25 (12)
Anti-nucleosome	44 (21)
Anti-histone	42 (20)
Antiphospholipid syndrome, *n* (%)	37 (17)
Antiphospholipid autoantibodies, *n* (%)	69 (32)
Lupus anticoagulant, *n* (%)	48 (23)
ACA IgM, *n* (%)	29 (14)
ACA IgG, *n* (%)	29 (14)
Anti beta2 glycoprotein IgM, *n* (%)	20 (10)
Anti beta2 glycoprotein IgG, *n* (%)	21 (10)
Current prednisone, *n* (%)	74 (35)
Prednisone, mg/day	5 (2.5–5)
Hydroxychloroquine, *n* (%)	154 (72)
Methotrexate, *n* (%)	28 (13)
Mycophenolate mofetil, *n* (%)	26 (12)
Azathioprine, *n* (%)	20 (9)
Rituximab, *n* (%)	5 (2)
Belimumab, *n* (%)	27 (13)

Data represent means ± SD or median (interquartile range) when data were not normally distributed. BMI: body mass index; C3 C4: complement; CRP: C-reactive protein; LDL: low-density lipoprotein; DMARD: disease-modifying antirheumatic drug; ACA: anticardiolipin; HDL: high-density lipoprotein; ANA: antinuclear antibodies; ENA: extractible nuclear antibodies; LLDAS: Low Lupus Disease Activity State; DORIS: Definitions Of Remission In SLE; SLEDAI: Systemic Lupus Erythematosus Disease Activity Index; SLEDAI categories were defined as follows: 0 no activity, 1–5 mild activity, 6–10 moderate activity, >10 high activity, >20 very high activity; SLICC: Systemic Lupus International Collaborating Clinics/American College of Rheumatology Damage Index.

**Table 2:** Multivariable analysis of disease-related data association with 3-nitrotyrosine.

	1/3-Nitrotyrosine × 1000, ng/mL			
	Beta Coef. (95% CI), *p*			
	Univariable		Multivariable	
Age, years	0.06 (−0.72–0.84)	0.88		
Female	−22 (−53–10)	0.18		
Body mass index, kg/m2	**1.53 (0.03–3.03)**	**0.046**		
Abdominal circumference, cm	0.42 (−0.21–1.06)	0.19		
Waist circumference, cm	0.51 (−0.12–1.14)	0.16		
Waist to hip ratio	−0.55 (−28.82–27.72)	0.97		
**Cardiovascular co-morbidity**				
Smoking	4 (−19–29)	0.69		
Diabetes	−16 (−58–26)	0.45		
Hypertension	2 (−17–21)	0.86		
Obesity	**21 (0.42–42)**	**0.046**		
Dyslipidemia	0.59 (−18.94–20.12)	0.95		
Statins	5 (−16–26)	0.65		
Aspirin	−3 (−24–18)	0.81		
**SLE-related data**				
Disease duration, years	**1.04 (0.21–1.87)**	**0.015**	**0.93 (0.09–1.77)**	**0.030**
CRP, mg/dl	−0.059 (−0.19–0.08)	0.40		
SLICC-DI	**11 (4–18)**	**0.002**	**10 (3–17)**	**0.006**
SLICC-DI >=1	**20 (11–48)**	**0.002**	**27 (9–45)**	**0.004**
SLEDAI−2K	**6 (2–10)**	**0.001**	**6. (3–10)**	**0.001**
SLEDAI categories				
No activity	ref.		Ref.	
Mild	**28 (8–47)**	**0.006**	**27 (8–47)**	**0.006**
Moderate to very high	**46 (14–78)**	**0.005**	**46 (15–78)**	**0.004**
DORIS	**−30 (−50–(−11))**	**0.002**	**−28 (−47–(−9))**	**0.004**
LLDAS	**−30 (−49–(−9))**	**0.004**	**−27 (−47–(−7))**	**0.008**
Auto-antibody profile				
Anti-DNA positive	20 (−0.75–42)	0.059	18 (−3–40)	0.089
Anti-ENA positive	**26 (5–47)**	**0.013**	**27 (6–47)**	**0.011**
Anti-SSA	7 (−12–27)	0.73		
Anti-SSB	18 (−8–45)	0.17	21 (−5–47)	0.12
Anti-Sm	17 (−7–41)	0.16	14 (−10–38)	0.26
Anti-RNP	**26 (6–46)**	**0.011**	**25 (5–46)**	**0.013**
Anti-ribosome	**36 (7–64)**	**0.014**	**36 (8–64)**	**0.012**
Anti-nucleosome	**31 (8–54)**	**0.008**	**31 (8–53)**	**0.008**
Anti-histone	13 (−10–36)	0.28		
Antiphospholipid syndrome	−8 (−33–16)	0.50		
Antiphospholipid autoantibodies	−12 (−34–10)	0.29		
Lupus anticoagulant	**−29 (−51–(−7))**	**0.010**	**−34 (−56–(−13))**	**0.002**
ACA IgM	−12 (−39–14)	0.36		
ACA IgG	4 (−23–31)	0.79		
Anti beta2 glycoprotein IgM	−13 (−44–19)	0.43		
Anti beta2 glycoprotein IgG	2 (−29–33)	0.91		
Current prednisone	12 (−8–31)	0.24		
Prednisone, mg/day	2 (−3–6)	0.54		
Hydroxychloroquine	−1 (−21–20)	0.96		
Methotrexate	4 (−23–32)	0.76		
Mycophenolate mofetil	−4 (−33–25)	0.77		
Azathioprine	11 (−21–42)	0.51		
Rituximab	14 (−48–75)	0.44		
Belimumab	22 (−5–50)	0.11	25 (−3–53)	0.077

In this analysis, the serum levels of 3-NT are the dependent variable. The multivariable analysis is adjusted for sex and body mass index. SLEDAI: Systemic Lupus Erythematosus Disease Activity Index; LLDAS: Low Lupus Disease Activity State; DORIS: Definitions Of Remission In SLE; the SLEDAI categories were defined as follows: 0 no activity, 1–5 mild activity, 6–10 moderate activity, >10 high activity, >20 very high activity; SLICC: Systemic Lupus International Collaborating Clinics/American College of Rheumatology Damage Index; CRP: C-reactive protein; ENA: extractable autoantibodies; ACA: anticardiolipin autoantibodies. Significant *p* values are depicted in bold.

**Table 3:** Multivariable analysis of cardiovascular disease-related factors’ association with 3-nitrotyrosine.

	1/3-Nitrotyrosine × 1000, ng/mL			
	Beta coef. (95% CI), *p*			
	Univariable		Multivariable	
Lipid profile				
Cholesterol, mg/dL	0.2 (−0.058–0.42)	0.14	0.2 (−0.04–0.4)	0.097
Triglycerides, mg/dL	**0.2 (0.06–0.4)**	**0.009**	**0.2 (0.03–0.4)**	**0.024**
HDL-cholesterol, mg/dL	−0.6 (−1.2–0.09)	0.089	−0.5 (−1–0.2)	0.17
LDL-cholesterol, mg/dL	0.2 (−0.05–0.5)	0.14	0.2 (−0.02–0.5)	0.070
LDL:HDL-cholesterol ratio	**16 (5–27)**	**0.007**	**15 (4–27)**	**0.009**
Non-HDL-cholesterol, mg/dL	**0.3 (0.04–0.6)**	**0.023**	**0.3 (−0.05–0.6)**	**0.021**
Lipoprotein A, mg/dL	0.02 (−0.1–0.2)	0.70		
Apolipoprotein A1, mg/dL	−0.3 (−0.6–0.06)	0.11	−0.2 (−0.6–0.09)	0.15
Apolipoprotein B, mg/dL	**0.5 (0.06–0.9)**	**0.025**	**0.5 (0.05–0.9)**	**0.029**
Apo B:Apo A1 ratio	**80 (27–134)**	**0.004**	**76 (22–130)**	**0.006**
Atherogenic index	**15 (6–24)**	**0.001**	**14 (5–23)**	**0.002**
**Insulin resistance indices**				
Glucose, mg/dL	0.1 (−0.4–0.7)	0.65		
C-peptide, ng/mL	2 (−4–7)	0.59		
Insulin, µU/mL	0.2 (−0.4–0.8)	0.57		
HOMA2-IR	2 (−4–7)	0.53		
HOMA2-S%	−0.005 (−0.1–0.1)	0.93		
HOMA2-B%-C-peptide	−0.01 (−0.2–0.2)	0.91		
**Carotid ultrasound**				
cIMT, microns	−0.3 (−0.1–0.05)	0.44		
Carotid plaque	15 (−6–36)	0.17	14 (−7–36)	0.20
**SCORE2 calculator**	**0.4 (−2–3)**	**0.77**		
Low risk	ref.		ref.	
Moderate risk	−0.5 (−28–27)	0.97	−6 (−34–22)	0.67
High risk	**44 (6–83)**	**0.025**	37 (−2–76)	0.060

In this analysis, serum levels of 3-NT are dependent variable. HOMA2-IR: insulin resistance index through homeostatic model assessment (calculated with glucose and insulin serum levels). HOMA2-S%: insulin sensitivity index through homeostatic model assessment (calculated with glucose and insulin serum levels). HOMA2-B%-C-peptide: β-cell function index through homeostatic model assessment (calculated with glucose and C-peptide serum levels). SCORE2: Systematic Coronary Risk Assessment; high density lipoprotein; LDL: low density lipoprotein; cIMT: carotid intima–media thickness. Multivariable analysis is adjusted for sex and body mass index. Significant *p* values are depicted in bold.

## Data Availability

Data will be made available on request.

## Supplementary Materials

The following supporting information can be downloaded at: https://www.mdpi.com/article/10.3390/antiox14060739/s1; Table S1: Relation of SDI score items to 3-NT serum values; Table S2: SLEDAI−2k items’ relation to 3-NT.

## Abbreviations

The following abbreviations are used in this manuscript:

3-NT	3-Nitrotyrosine
SLE	Systemic lupus erythematosus
CV	Cardiovascular
CVD	Cardiovascular disease
ACR	American College of Rheumatology
SLEDAI−2K	Systemic Lupus Erythematosus Disease Activity Index
SLICC-SDI	Systemic Lupus International Collaborating Clinics/American College of Rheumatology Damage Index
DORIS	Definitions of Remission in SLE
LLDAS	Lupus Low Disease Activity State
PGA	Physician global assessment
SCORE2	Systematic Coronary Risk Evaluation−2
HDL	High-density cholesterol
SCORE2-OP	Systematic Coronary Risk Evaluation−2 Older Persons
LDL	Low-density cholesterol
HOMA	Homeostatic model assessment
IR	Insulin resistance
cIMT	Carotid intima–media
SD	Standard deviation
IQR	Interquartile range
BMI	Body mass index
CRP	C-reactive protein
ACAs	Anticardiolipin antibodies
ANAs	Antinuclear antibodies
ENAs	Extractible nuclear antibodies
DMARD	Disease-modifying antirheumatic drug
ACVA	Acute cerebrovascular accident
