# Supplementary material for: Serum 3-Nitrotyrosine in the Cardiovascular Disease of Patients with Systemic Lupus Erythematosus
Source: Antioxidants (Basel). 2025 Jun 16;14(6):739. doi: 10.3390/antiox14060739 (PMC12189452; doi:10.3390/antiox14060739)
Supplement: Supplementary file 1 [file antioxidants-14-00739-s001.zip › antioxidants-3589274-supplementary.pdf]

Supplementary Table S1. Relation of SDI score items to 3-NT serum values

			1/3-Nitrotyrosine x 1000, ng/ml	
	n	%	beta coef. (95%)	p
**Ocular**				
Any cataract ever	23	11	**33 (3-64)**	**0.030**
Retinal change or optic atrophy	28	13	10 (-18-37)	0.49
Points =>1 in the domain	48	22	**21 (-1-43)**	**0.006**
**Neuropsychiatric**				
Cognitive impairment	1	1	-14 (-150-122)	0.84
Seizures requiring therapy for 6 months	4	2	36 (-32-104)	0.30
Cerebrovascular accident ever	1	1	-39 (-94-17)	0.17
Cranial or peripheral neuropathy	1	1	**140 (5-274)**	**0.042**
Transverse myelitis	2	1	-6 (-102-91)	0.91
Points =>1 in the domain	10	5	13 (-31-57)	0.57
**Renal**				
Estimated or measured glomerular filtration rate <50%	8	4	-14 (-63-35)	0.58
Proteinuria 3.5 gm/24 hours	0	0	-	
End-stage renal disease	2	1	16 (-80-113)	0.33
Points =>1 in the domain	10	5	-8 (-52-36)	0.73
**Pulmonary**				
Pulmonary hypertension	2	1	8 (-88-104)	0.87
Pulmonary fibrosis	2	1	68 (-28-164)	0.16
Shrinking lung	0	0	-	
Pleural fibrosis	0	0	-	
Pulmonary infarction	0	0	-	
Points =>1 in the domain	3	1	29 (-50-108)	0.47
**Cardiovascular**				
Angina or coronary artery bypass	0	0	-	
Myocardial infarction ever	4	2	44 (-34-123)	0.27
Cardiomyopathy	1	1	89 (-54-217)	0.24
Valvular disease	5	2	25 (-43-94)	0.47
Pericarditis for 6 months, or pericardiectomy	6	3	**69 (13-124)**	**0.015**
Points =>1 in the domain	14	7	**58 (20-96)**	**0.003**
**Peripheral vascular**				
Claudication for 6 months	0	0	-	
Minor tissue loss (pulp space)	1	1	38 (-98-179)	0.58
Significant tissue loss ever	0	0	-	
Venous thrombosis	14	7	-12 (-49-26)	0.55
Points =>1 in the domain	15	7	-8 (-44-28)	0.66
**Gastrointestinal**				
Infarction or resection of bowel	2	1	23 (-74-119)	0.64
Mesenteric insufficiency	0	0	-	
Chronic peritonitis	0	0	-	
Stricture or upper gastrointestinal tract surgery ever	0	0	-	
Pancreatic insufficiency	0	0	-	
Points =>1 in the domain	2	1	23 (-74-119)	0.64
**Musculoskeletal**				
Muscle atrophy or weakness	0	0	-	
Deforming or erosive arthritis	26	12	**41 (13-69)**	**0.004**
Osteoporosis with fracture or vertebral collapse	9	4	12 (-34-59)	0.60
Avascular necrosis	9	4	13 (-33-59)	0.58

Osteomyelitis	0	0	-	
Tendon rupture	5	2	3 (-58-65)	0.92
Points =>1 in the domain	46	22	**33 (11-55)**	**0.004**
**Skin**				
Scarring chronic alopecia	7	3	**102 (47-156)**	**0.000**
Severe scarring or thickening of the panniculus	9	4	29 (-17-75)	0.21
Skin ulceration	2	1	27 (-70-123)	0.59
Points =>1 in the domain	18	8	**57 (24-91)**	**0.001**
**Premature gonadal failure**	**18**	**9**	**12 (-21-46)**	**0.48**
**Diabetes (regardless of treatment)**	**7**	**3**	**-11 (-63-41)**	**0.69**
**Malignancy (exclude dysplasia)**	**11**	**5**	**6 (-36-48)**	**0.78**

Significant p values are depicted in bold.

Supplementary Table S2. SLEDAI-2k items relation to 3-NT

	n	%	1/3-Nitrotyrosine x 1000, ng/ml<br>beta coef. (95%)	p
Seizures	0	0		
Psychosis	1	0	-2 (-19-15)	0.84
Organic brain syndrome	0	0		
Visual disturbance	0	0		
Cranial nerve disorder	0	0		
Lupus headache	0	0		
ACVA	0	0		
Vasculitis	0	0		
Arthritis	14	7	4 (-6-14)	0.41
Myositis	0	0		
Urinary cylinders	0	0		
Hematuria	5	2	13 (-4-30)	0.15
Proteinuria	7	3	4 (-10-18)	0.56
Pyuria	3	1	26 (-53-104)	0.52
Rash	24	11	**15 (0.6-30)**	**0.042**
Alopecia	9	4	14 (-9-37)	0.22
Mucosal ulcers	19	9	9 (-7-26)	0.25
Pleurisy	1	0	-31 (-99-37)	0.37
Pericarditis	0	0		
Low complement	62	29	15 (-6-36)	0.15
Elevated anti-DNA	61	30	**21 (1-42)**	**0.040**
Fever	2	1	7 (-3-17)	0.18
Thrombopenia	5	2	17 (-44-78)	0.59
Leukopenia	16	7	3 (-32-38)	0.86

ACVA: Acute Cerebrovascular Accident. Significant p values are depicted in bold.
